# Supplementary material for: Analysis of the Promoter Regions of gga-miR-31 and Its Regulation by RA and C-jun in Chicken
Source: Int J Mol Sci. 2023 Aug 7;24(15):12516. doi: 10.3390/ijms241512516 (PMC10420004; doi:10.3390/ijms241512516)
Supplement: Supplementary file 1 [file ijms-24-12516-s001.zip › ijms-2444917-supplementary.pdf]

Table S1. Information of different species miR-31 in phylogenetic tree

Gene	Species	Number
gga-miR-31	Chicken	NR_031499.1
hsa-miR-31	Human	NR_029505.1
mmu-miR-31	House Mouse	NR_029747.1
bmo-miR-31	Domenstic Silkworm	NR_107250.1
cfa-miR-31	Dog	NR_049225.1
eca-miR-31	Horse	NR_032994.1
tgu-miR-31	Zebra finch	NR_049153.1
bta-miR-31	Cattle	NR_049716.1
dre-miR-31	Zebra fish	NR_034276.1
ola-miR-31	Japanese Medaka	NR_107194.1
ptr-miR-31	Chimpanzee	NR_032004.1
ssc-miR-31	Pig	NR_128414.1
ocu-miR-31	Rabbit	NR_162489.1
cgr-miR-31	Chinese hamster	NR_105204.1
mml-miR-31	Rhesus Monkey	NR_032003.1
rno-miR-31	Norway Rat	NR_031845.1

Table S2. Primers sequences of gga-miR-31-5p promoter different recombinant vectors

Name of fragment	Length of product	Primer sequence（5'-3'）
pGL3-miR-31-2180	2226bp	F2:atttctctatcgataggtaccCCTGCAAGGTCACAGTGAAAC<br>R2:gcttacttagatcgcagatctcgagCTTGTTAGAAAGCCATCTG
pGL3-miR-31-1344	1390bp	F3:atttctctatcgataggtaccAAGCTGAACTTCATACAC<br>R2:gcttacttagatcgcagatctcgagCTTGTTAGAAAGCCATCTG
pGL3-miR-31-584	630bp	F4:atttctctatcgataggtaccGATCACTTACAGCATGCTG<br>R2:gcttacttagatcgcagatctcgagCTTGTTAGAAAGCCATCTG
Fragment 1	213bp	dF1:atttctctatcgataggtaccCCTGCAAGGTCACAGTGAAAC<br>dR1:caagtctacaATTAACACATTAAAGCCTTTCCCTC
Fragment 2	2023bp	dF2:atgtgttaatTGTAGACTTGTTTGTTGTC<br>dR2: gcttacttagatcgcagatctcgagCTTGTTAGAAAGCCATCTG
Fragment 3	405bp	dF1: atttctctatcgataggtaccCCTGCAAGGTCACAGTGAAAC<br>dR3: catctgttgaTTCTGATTAAGTAGAAAAATTAATTGTAG
Fragment 4	1831bp	dF3: ttaatcagaaTCAACAGATGGTTTGCTTTG<br>dR2: gcttacttagatcgcagatctcgagCTTGTTAGAAAGCCATCTG
Fragment 5	202bp	dF2: atgtgttaatTGTAGACTTGTTTGTTGTC<br>dR3: catctgttgaTTCTGATTAAGTAGAAAAATTAATTGTAG

Note: The lowercase letters are homologous arms.

Table S3. Prediction of transcription factors in the gga-miR-31-5p promoter region

Promoter region	Transcription factor
-584bp~+1bp	Ap-1, Atf, C/Ebpalpha, C/Ebpbeta, Cp1, CREB, CRE-BP1, Dl, Ftz, GATA-1, GR, Hb, HNF-3, HSF1_(Long), ICSBP, MEB-1, NF-1, Oct-1, Oct-2.1, P40x, RAP1, Sp1, SRF, TBP, Zen-1
-1344bp~-584bp	C/EBPalpha, Antp, AP-1, GCN4, C/EBPgamma, Sp1, GR, PR, NF-1, C/EBP, SRF, TBP, IRF-1, ADR1, CRE-BP1, GATA-1, ATF-a, Oct-1, Odd, C/EBPbeta, CPE_binding_pro, CREB, MEB-1, GCR1, C/EBPdelta, GLO, Hb, Ftz, Pit-1, HOXA4
-2180bp~-1344bp	HNF-4alpha1, GR, c-Jun, CFF, HSTF, NF-E2, ER, Oct-1, NF-1, RAR-alpha1, C/EBPepsilon, MEB-1, ICSBP, Antp, C/EBPalpha, PR, TBP, Oct-11, Pit-1a, RAP1, HNF-3, Sp1, C/EBPbeta, SRF, HNF-1C, GCN4, GATA-1, HNF-1, REB1, REV-ErbAalpha, Odd, T3R-alpha, CPE_binding_pro, C/EBPgamma, LyF-1, Oct-2.1, ETF, PU.1
